# Autophagy is involved in TGF-β1-induced protective mechanisms and formation of cancer-associated fibroblasts phenotype in tumor microenvironment

**DOI:** 10.18632/oncotarget.6702

**Published:** 2015-12-21

**Authors:** Fang-Lan Liu, En-Pan Mo, Liu Yang, Jun Du, Hong-Sheng Wang, Huan Zhang, Hiroshi Kurihara, Jun Xu, Shao-Hui Cai

**Affiliations:** ^1^ Pharmacy College, Jinan University, Guangzhou 510632, China; ^2^ Pharmacy College, Sun Yat-Sen University, Guangzhou 510405, China

**Keywords:** autophagy, TGF-β1, tumor microenvironment, mitochondria, cancer-associated fibroblasts

## Abstract

Transforming growth factor-β1 (TGF-β1) present in tumor microenvironment acts in a coordinated fashion to either suppress or promote tumor development. However, the molecular mechanisms underlying the effects of TGF-β1 on tumor microenvironment are not well understood. Our clinical data showed a positive association between TGF-β1 expression and cancer-associated fibroblasts (CAFs) in tumor microenvironment of breast cancer patients. Thus we employed starved NIH3T3 fibroblasts *in vitro* and 4T1 cells mixed with NIH3T3 fibroblasts xenograft model *in vivo* to simulate nutritional deprivation of tumor microenvironment to explore the effects of TGF-β1. We demonstrated that TGF-β1 protected NIH3T3 fibroblasts from Star-induced growth inhibition, mitochondrial damage and cell apoptosis. Interestingly, TGF-β1 induced the formation of CAFs phenotype in starvation (Star)-treated NIH3T3 fibroblasts and xenografted Balb/c mice, which promoted breast cancer tumor growth. In both models, autophagy agonist rapamycin increased TGF-β1-induced protective effects and formation of CAFs phenotypes, while autophagy inhibitor 3-methyladenine, *Atg5* knockdown or TGF-β type I receptor kinase inhibitor LY-2157299 blocked TGF-β1 induced these effects. Taken together, our results indicated that TGF-β/Smad autophagy was involved in TGF-β1-induced protective effects and formation of CAFs phenotype in tumor microenvironment, which may be used as therapy targets in breast cancer.

## INTRODUCTION

Tumor microenvironment has emerged as an important target for cancer therapy. For most solid tumors, particularly carcinomas, their microenvironment consists of the tumor cells themselves, endothelial cells, immune cells and fibroblasts contribute to tumorigenesis by secretion of cytokines and/or direct cell-cell contact [1]. Importantly, the different cell types within the tumor microenvironment communicate both between themselves and with each other in order to support tumor growth [2]. In particular, a subpopulation of fibroblasts, the so-called cancer-associated fibroblasts (CAFs), seems to activate many aspects of tumorigenesis. CAFs are a subpopulation of fibroblasts found in the tumor microenvironment [3], They are myofibroblasts, or activated fibroblasts in the tumor stroma, mostly characterized by the expression of activated fibroblast markers, such as α-smooth muscle actin (α-SMA) and fibroblast activation protein-α (FAP-α) [4]. It is widely assumed that myofibroblasts may also develop from NIH3T3 fibroblasts, as both cell types show more similarities than differences including the expression of cytoskeleton proteins like FAP-α and α-SMA [5]. Many researchers have reported that CAFs facilitated tumor initiation, progression, and metastasis [6–8]. Moreover, it is reported that FAP-α and calponin can serve as a novel marker for pathologically diagnosing the existence of microinvasion in ductal carcinoma in situ (DCIS) [9]. Emerging study indicates that transforming growth factor-β1 (TGF-β1), a multifunctional cytokine that regulates the growth, differentiation and migration of various types of cells [10], has been recognized as the most potent inducer for the transformation of fibroblasts to CAFs [11]. Moreover, several studies have demonstrated that TGF-β1 is capable of activating α-SMA-negative tumor stromal fibroblasts into α-SMA-positive CAFs [12, 13]. However, mechanisms for CAFs activation by TGF-β in tumor microenvironment are not well understood.

Autophagy is a bulk lysosomal degradation pathway that mediates the clearance of cytoplasmic components including macromolecules (for example proteins, glycogens, lipids and nucleotides) and organelles (for example mitochondria, peroxisomes and endoplasmic reticulum) [14]. During the initiation of autophagy, a family of autophagy regulatory proteins activates the formation of autophagosome. ATG5–ATG12 complex and LC3 play a predominant role in the formation of pre-autophagosome [15]. Another autophagy regulatory protein Beclin 1 functions for localization of autophagic proteins to a pre-autophagosomal structure. Beclin1-induced autophagosome formation is controlled by the inhibition of mTOR pathway and class-III pathway (Atg6)/hVps34 [16, 17]. Autophagy is an internal course of catabolism which is essential for cell growth, development and cellular homeostasis under various stress conditions including nutrient deprivation, growth factor depletion, and hypoxia [18–21]. Moreover, It has been reported that autophagy in certain tumor types including breast cancer might be thus implicated in tumor promotion in the later phase of tumorigenesis. Nevertheless, tumors are the ecosystems of cancer cells and their stroma coevolution [22]. Recently, the primary research focus on autophagy in cancer has shifted from cancer cell-centric to tumor stroma. Previous studies have reported that autophagy in tumor stroma promoted tumor growth and progression in conditions of hypoxia and metabolic stress [23–25]. However, the function of autophagy in tumor microenvironment is complicated, and its mechanisms are still ambiguous.

Emerging studies have indicated that TGF-β1 might be a potent activator of autophagy [26, 27]. The pro-autophagy effects of TGF-β1 have been well demonstrated in various cell types, including in normal bovine mammary epithelial BME-UV1 cells [28], mouse mesangial cells [29] and hepatocellular carcinoma [30]. Moreover, TGF-β1-induced autophagy is required for the fibrogenic response in human atrial myofibroblasts [26]. However, whether autophagy is involved in TGF-β1 induced effects on tumor microenvironment are not well understood.

To address these issues, we utilized a vitro model with NIH3T3 mouse embryonic fibroblasts challenged with serum starvation (Star), to investigate TGF-β1 induced effects on starved NIH3T3 fibroblasts. Meanwhile, we employed a mixed xenograft as a comparable *in vivo* model to investigate TGF-β1 induced effects on tumor microenvironment and solid tumor survival and growth.

## RESULTS

### Expression of TGF-β and CAFs maker α-SMA were both increased in tumor tissues of breast cancer patients

**Figure 1:**
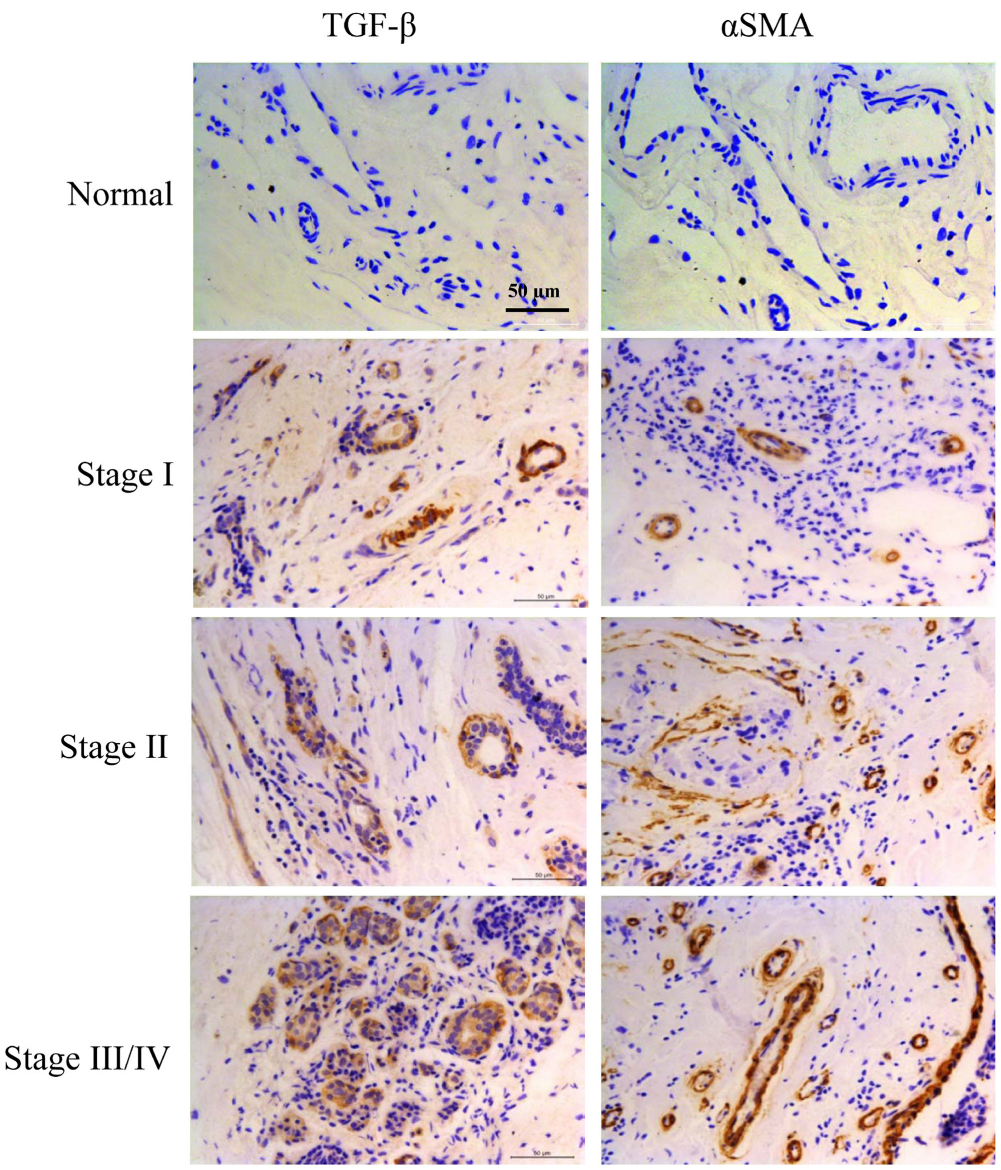
Expression of TGF-β and CAFs maker α-SMA were both increased in tumor tissues of breast cancer patients TGF-β and α-SMA expression were analyzed by immunohistochemistry staining in normal breast tissue (n=10) or tumor tissues from patients with breast cancer (n=121).

To investigate the relationship between TGF-β1 and CAFs in tumor microenvironment, we detected the expression of TGF-β and CAFs maker α-SMA in normal breast tissue and tumor tissues obtained from patients with clinical stage I–IV breast cancer. Our results showed that a minimum expression of TGF-β and α-SMA in the normal breast tissue (n=10), while they were obviously increased in tumor tissues (n=121), especially from the samples of patients with clinical stage III/IV breast cancer (Figure 1). The results revealed a positive association between TGF-β expression and CAFs in tumor microenvironment of breast cancer patients.

### TGF-β1 exerted protective effects and induced formation of CAFs phenotype in Star-treated NIH3T3 fibroblasts

**Figure 2:**
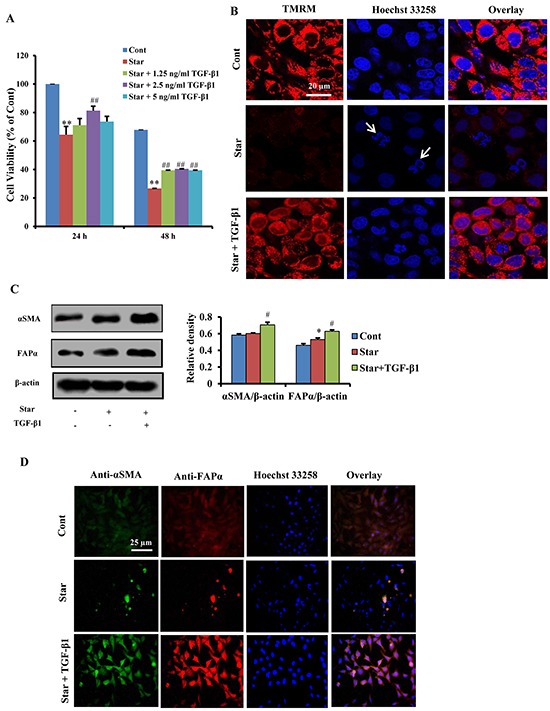
TGF-β1 exerted protective effects and induced formation of CAFs phenotype in Star-treated NIH3T3 fibroblasts **A.** Time-dependent growth inhibitory effects of Star in NIH3T3 cells. Cells were first incubated under serum-free condition for different time (24∼48 h), and then treated with different concentrations TGF-β1 (1.25ng/ml∼5ng/ml) in 10% fetal bovine serum for 24 h. The inhibitory ratio was determined by MTT assay. **B.** Cells were first incubated under serum-free condition for 24 h, and then treated with TGF-β1 (2.5ng/ml) in 10% fetal bovine serum for 24 h. MMP was evaluated with TMRM staining using confocal laser scanning microscopy. Cells were counter-stained with Hoechest 33258 for DNA. Arrows indicate cells with poor shape. **C.** Cells were first incubated under serum-free condition for 24 h, and then treated with TGF-β1 (2.5ng/ml) in 10% fetal bovine serum for 24 h. Cells were subjected to western blotting analysis with antibodies directed against CAFs markers α-SMA and FAP-α. β-actin was used as an equal loading control. **D.** Cells were first incubated under serum-free condition for 24 h, and then treated with TGF-β1 (2.5ng/ml) in 10% fetal bovine serum for 24 h. Fluorescence microscopy of NIH3T3 fibroblasts dually stained with α-SMA (probed with primary anti-α-SMA antibody, and a secondary antibody using FITC 488, Santa Cruz, USA) and FAP-α (probed with primary anti-FAP-α antibody and a secondary antibody using DyLight 649, Abbkine, USA). Cells were counter-stained with Hoechest 33258 for DNA. Cont, control; Star: starvation, free-serum for 24 h; TGF-β1, 2.5 ng/ml; Rapa, 500 nM; 3-MA, 2 Mm. Data were expressed as the means ± S.E.M. (n=3). ^**^P < 0.01 vs. Cont, ^#^*P* < 0.05 and ^##^*P* < 0.01 vs. Star-treated cells.

To simulate the nutritional deprivation of tumor microenvironment, we utilized NIH3T3 mouse embryonic fibroblasts challenged with Star as an *in vitro* model. MTT assay showed that Star significantly inhibited cell proliferation after serum-free incubation for 24 h or 48 h (P < 0.01). The growth inhibition induced by Star was significantly attenuated by TGF-β1 (1.25-5 ng/ml), especially at 2.5 ng/ml (P < 0.01) (Figure 2A). Mitochondrial membrane potential (MMP) has been proposed as an ideal biomarker for environmental stress [31]. Thus, we evaluated the level of MMP with TMRM staining using confocal laser scanning microscopy. Our results demonstrated that Star resulted in a loss of MMP in NIH3T3 fibroblasts. TGF-β1 treatment (2.5 ng/ml) relieved Star-induced loss of MMP. Using Hoechst staining, we observed an increased DNA fragmentation (a hallmark of apoptosis) in Star-treated NIH3T3 cells. The decreased DNA fragmentation found in TGF-β1-treated cells suggested a protective role of TGF-β1 (Figure 2B). In addition, western blotting analysis showed that TGF-β1 induced CAFs features in Star-treated NIH3T3 fibroblasts, which was characterized with positive expression of α-SMA and FAP-α (Figure 2C). The CAFs features were further confirmed by the results of immunofluorescent microscopy (Figure 2D).

### TGF-β1 enhanced autophagy in Star-treated NIH3T3 fibroblasts

**Figure 3:**
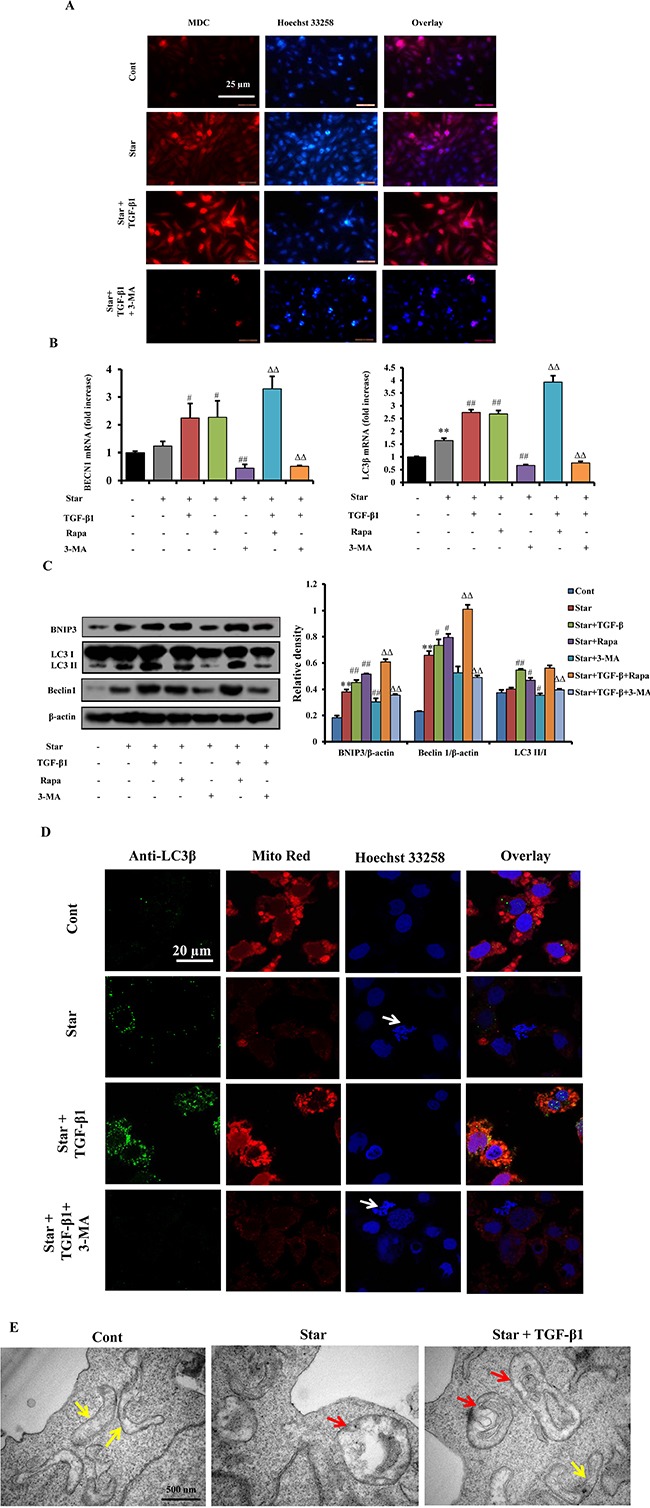
TGF-β1 enhanced autophagy in Star-treated NIH3T3 fibroblasts Cells were first incubated under serum-free condition for 24 h, and then treated with TGF-β1 (2.5ng/ml) in 10% fetal bovine serum and the presence or absence of Rapa (500 nM) or 3-MA (2 mM) for 24 h. **A.** Formation of autophagic vacuoles was evaluated with MDC staining using immunofluorescence. **B.** Autophagy related genes of MAPLC3β and BECN1 were analyzed by Q-PCR. **C.** Protein expression levels of the mitophagy (BNIP3) and autophagy markers (Beclin-1 and LC3-II/I conversion) were evaluated by western blotting. Cells were first incubated under serum-free condition for 24 h, and then treated with TGF-β1 (2.5ng/ml) in 10% fetal bovine serum and the presence or absence of Rapa (500 nM) or 3-MA (2 mM) for 24 h. **D.** Confocal fluorescence microscopy of NIH3T3 fibroblasts dually stained with LC3β (probed with primary anti-LC3β antibody, and a secondary antibody using Alexa Fluor, Cell Signaling Technology) and Mito Red. Cells were counter-stained with Hoechest 33258 for DNA. Arrows indicate cells with poor shape. **E.** Transmission electron microscopy of NIH3T3 fibroblasts. Autolysosomes were indicated with red arrows; swelling mitochondria were indicated with yellow arrows. Cont, control; Star: starvation, free-serum for 24 h; TGF-β1, 2.5 ng/ml; Rapa, 500 nM; 3-MA, 2 mM. Data are expressed as the means ± S.E.M. (n=3). ^**^P < 0.01 vs. Cont, ^#^*P* < 0.05 and ^##^*P* < 0.01 vs. Star-treated cells, and ^ΔΔ^*P* < 0.01 vs. Star/TGF-β1 treated group.

To test whether TGF-β1 induced autophagy in Star-treated NIH3T3 fibroblasts, five different methods were employed to evaluate the level of autophagy. Firstly, MDC staining demonstrated that 24 h of serum-free incubation in NIH3T3 fibroblasts stimulated autophagy, as evidenced by the increased MDC positive ratio. Presence of TGF-β1 (2.5 ng/ml) increased the ratio of MDC staining in Star-stressed cells, while autophagy inhibitor 3-methyladenine (3-MA, 2 mM) blocked the effects of TGF-β1 (Figure 3A). Next, we demonstrated that treatment of cells with TGF-β1 significantly up-regulated the expression level of autophagy genes, including microtubule-associated protein *LC3β* (*MAPLC3β*) and *BECN1*, in Star-treated NIH3T3 fibroblasts. Rapa enhanced the effects of TGF-β1 on autophagy genes, which were suppressed by co-treatment of 3-MA (Figure 3B). The occurrence of autophagy was further confirmed by western blotting. Treatment of NIH3T3 cells with Star resulted in a slight accumulation of LC3-II. TGF-β1 treatment significantly enhanced LC3-II accumulation, with concomitant decrease of LC3-I. Protein expression of BNIP3 and Beclin 1 were also increased by TGF-β1 treatment. Consistent with the results of QPCR, the effects of TGF-β1 on Star-induced BNIP3 and Beclin 1 expression were enhanced by Rapa, while inhibited by 3-MA treatment (Figure 3C). The results of confocal fluorescence microscopy also revealed that TGF-β1 induced the accumulation of LC3β-II foci and increased the level of co-localization of LC3β puncta and mitochondria (MitoRed) in Star-treated NIH3T3 fibroblasts, suggesting the initiation of mitochondrial autophagy. In contrast, autophagy inhibitor 3-MA resulted in decrease of formation of LC3β-II foci (Figure 3D). Finally, the results of TEM demonstrated that control cells exhibited a normal tubular mitochondrial network (yellow arrows). In contrast, a deformation of mitochondrion was packaged by autophagosome (red arrows) in Star-treated cells. TGF-β1 increased the autophagic vacuole containing injured mitochondria and repaired the injured mitochondria in Star-treated NIH3T3 fibroblasts (Figure 3E).

### Autophagy was involved in TGF-β1-induced protection and CAFs phenotype formation in Star-treated NIH3T3 fibroblasts

**Figure 4:**
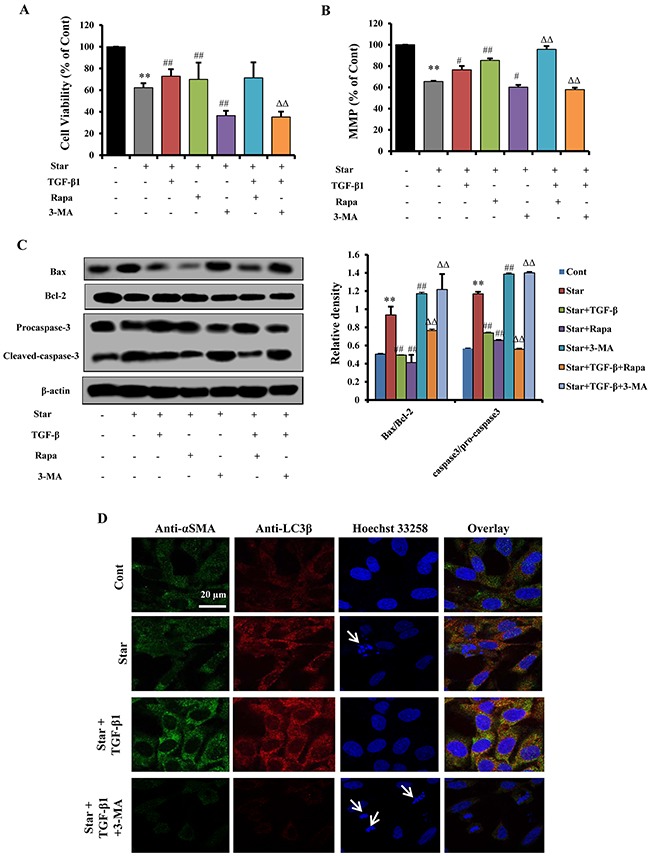
Autophagy was involved in TGF-β1-induced protection and CAFs phenotype formation in Star-treated NIH3T3 fibroblasts Cells were first incubated under serum-free condition for 24 h, and then treated with TGF-β1 (2.5ng/ml) in 10% fetal bovine serum and the presence or absence of Rapa (500 nM) or 3-MA (2 mM) for 24 h. **A.** Effect of TGF-β1 (2.5 ng/ml) and autophagy modulators (Rapa, 500 nM; 3-MA, 2 mM) on Star-induced growth inhibition. **B.** Evaluation of MMP with TMRM staining using flow cytometry. **C.** Apoptosis-related protein expression levels of caspase-3, caspase-9, Bax, and Bcl-2 in NIH3T3 fibroblasts were analyzed by western blotting. **D.** Confocal fluorescence microscopy of NIH3T3 fibroblasts dually stained with α-SMA (probed with primary anti-α-SMA antibody, and a secondary antibody using FITC 488, Santa Cruz, USA) and LC3β (probed with primary anti-LC3β antibody and a secondary antibody using DyLight 649, Abbkine, USA). Cells were counter-stained with Hoechest 33258 for DNA. Arrows indicate cells with poor shape. Cont, control; Star: starvation, free-serum for 24 h; TGF-β1, 2.5 ng/ml; Rapa, 500 nM; 3-MA, 2 mM. Data are expressed as the means ± S.E.M. (n=3). ^**^P < 0.01 *vs.* Cont, ^#^*P* < 0.05 and ^##^*P* < 0.01 *vs.* Star-treated cells, and ^ΔΔ^*P* < 0.01 *vs.* Star/ TGF-β1 treated group.

To understand whether autophagy was implicated in the beneficial effects of TGF-β1 in Star-treated NIH3T3 fibroblasts, further experiments were conducted. MTT assay showed that 2.5 ng/ml TGF-β1 significantly attenuated the growth inhibition in Star-treated NIH3T3 fibroblasts (P < 0.01). Meanwhile, this phenomenon was reinforced by Rapa (500 nM) and suppressed by 3-MA (2 mM) (Figure 4A). Then, we evaluated MMP with TMRM staining using flow cytometry. Our results demonstrated that TGF-β1 relieved Star-induced MMP loss in Star-treated NIH3T3 fibroblasts, while the effects of TGF-β1 was suppressed by 3-MA (Figure 4B). Moreover, a significant cleavage of caspase-3 was detected in Star-treated cells, and the protein expression of Bax was upregulated. TGF-β1 treatment could significantly protect NIH3T3 cells from nutrition deprivation. The effect of TGF-β1 was enhanced by Rapa while abolished by 3-MA treatment (Figure 4C). In addition, we investigated whether autophagy was involved in TGF-β1 induced CAFs features in Star-treated NIH3T3 fibroblast. A co-localization of α-SMA and LC3β-II puncta was detected by confocal fluorescence microscopy. Our results showed that the α-SMA and LC3β-II fluorescent spots were less in Cont group. The accumulation of LC3β-II foci were increased in cells treated with Star. The co-localization of α-SMA and LC3β-II puncta were promoted by TGF-β1 treatment, suggesting the involvement of autophagy in TGF-β1 induced formation of CAFs phenotype. In contrast, autophagy inhibitor 3-MA resulted in decrease of co-localization of α-SMA and LC3β-II foci (Figure 4D).

### *Atg5* knockdown blocked TGF-β1-induced protection and formation of CAFs phenotype in Star-treated NIH3T3 fibroblasts

**Figure 5:**
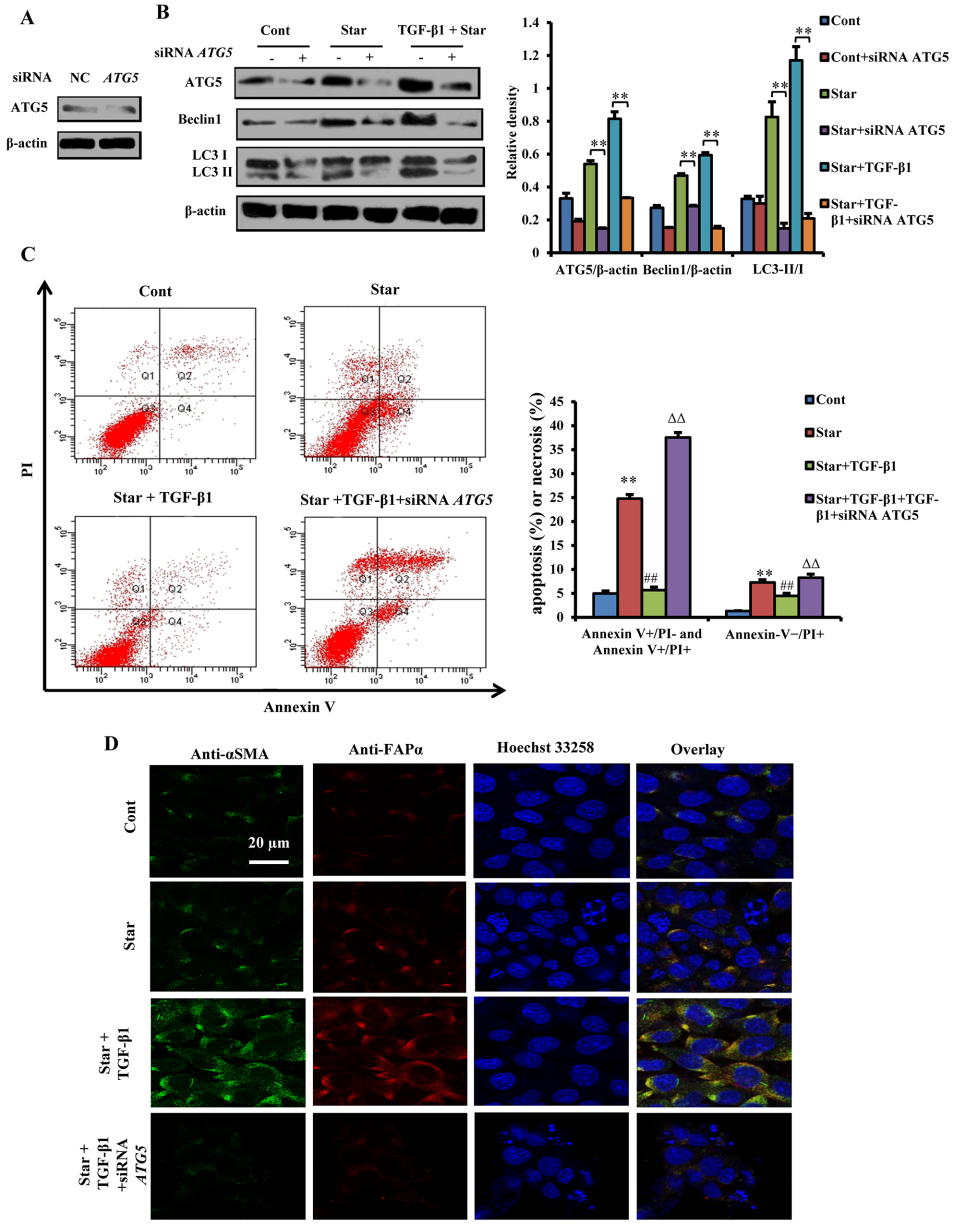
*Atg5* knockdown blocked TGF-β1-induced protection and formation of CAFs phenotype in Star-treated NIH3T3 fibroblasts *ATG5* knockdown was performed in NIH3T3 fibroblasts using RNAi procedure, *ATG5* siRNA transfected cells were first incubated under serum-free condition for 24 h, and then treated with 10% fetal bovine serum with TGF-β1 (2.5 ng/ml). **A.** *ATG5* knockdown was performed in NIH3T3 fibroblasts using RNAi procedure. **B.** Effect of *ATG5* knockdown on protein expression levels of ATG5, LC3-II/I, and Beclin 1 determined by western blotting. **C.** Cells were stained with fluorescein isothiocyanate (FITC)-conjugated annexin V (5 μg/ml) and PI (10 μg/ml). Cell apoptosis or necrosis was analyzed by flow cytometry. Positioning of quadrants on Annexin V/PI dot plots is performed, and living cells (Q3: Annexin V-/PI-), early apoptotic/primary apoptotic cells (Q4: Annexin V+/PI-), late apoptotic/secondary apoptotic cells (Q2: Annexin V+/PI+) and necrotic cells (Q1: Annexin V-/PI+) were distinguished. Total apoptotic proportion includes the percentage of cells with fluorescence Annexin V+/PI- and Annexin V+/PI+. **D.** Confocal fluorescence microscopy of NIH3T3 fibroblasts dually stained with α-SMA (probed with primary anti-α-SMA antibody, and a secondary antibody using FITC 488, Santa Cruz, USA) and FAP-α (probed with primary anti-FAP-α antibody and a secondary antibody using DyLight 649, Abbkine, USA). Cells were counter-stained with Hoechest 33258 for DNA. Cont, control; Star: starvation, serum-free for 24 h; TGF-β1, 2.5 ng/ml. Data are expressed as the means ± S.E.M. (n=3). ^**^P < 0.01 *vs.* Cont, ^#^*P* < 0.05 and ^##^*P* < 0.01 *vs.* Star-treated cells, and ^ΔΔ^*P* < 0.01 *vs.* Star/TGF-β1 treated group.

To confirm the function of autophagy in TGF-β1-induced protection and formation of CAFs phenotype in Star-treated NIH3T3 fibroblasts, RNA interference was utilized to knockdown *ATG5*, an important autophagy factor (Figure 5A). Results showed that *ATG5* knockdown had almost completely inhibited Star- or Star+TGF-β1-induced Beclin 1 expression and LC3β-II/I conversion (Figure 5B). Data also revealed that TGF-β1 protected NIH3T3 fibroblasts from Star-induced cell apoptosis and necrosis. According to their reactivity towards annexin V and PI, 93.73% of untreated NIH3T3 fibroblasts were viable (Annexin-V−/PI−). In contrast, 24 h of Star treatment increased the percentages of apoptotic cells, as well as that of necrotic cells. Meanwhile, we found that TGF-β1 protected NIH3T3 fibroblasts from Star-induced significant numbers of apoptotic and necrotic cells. However, *ATG5* knockdown could block the effect of TGF-β1 on apoptosis protection, resulting in the induction of Annexin V+/PI- and Annexin V+/PI+ apoptotic cells and Annexin-V−/PI+ necrotic cells in Star-treated NIH3T3 fibroblasts (Figure 5C). Interestingly, we also found that TGF-β1-induced expression of α-SMA and FAP-α was loss in *ATG5* knockdown cells, further confirming the involvement of autophagy in TGF-β1-induced formation of CAFs phenotype in Star-treated NIH3T3 fibroblasts (Figure 5D).

### TGF-β/Smad autophagy signaling pathway was involved in TGF-β1-induced protection and formation of CAFs phenotype in Star-treated NIH3T3 fibroblasts

**Figure 6:**
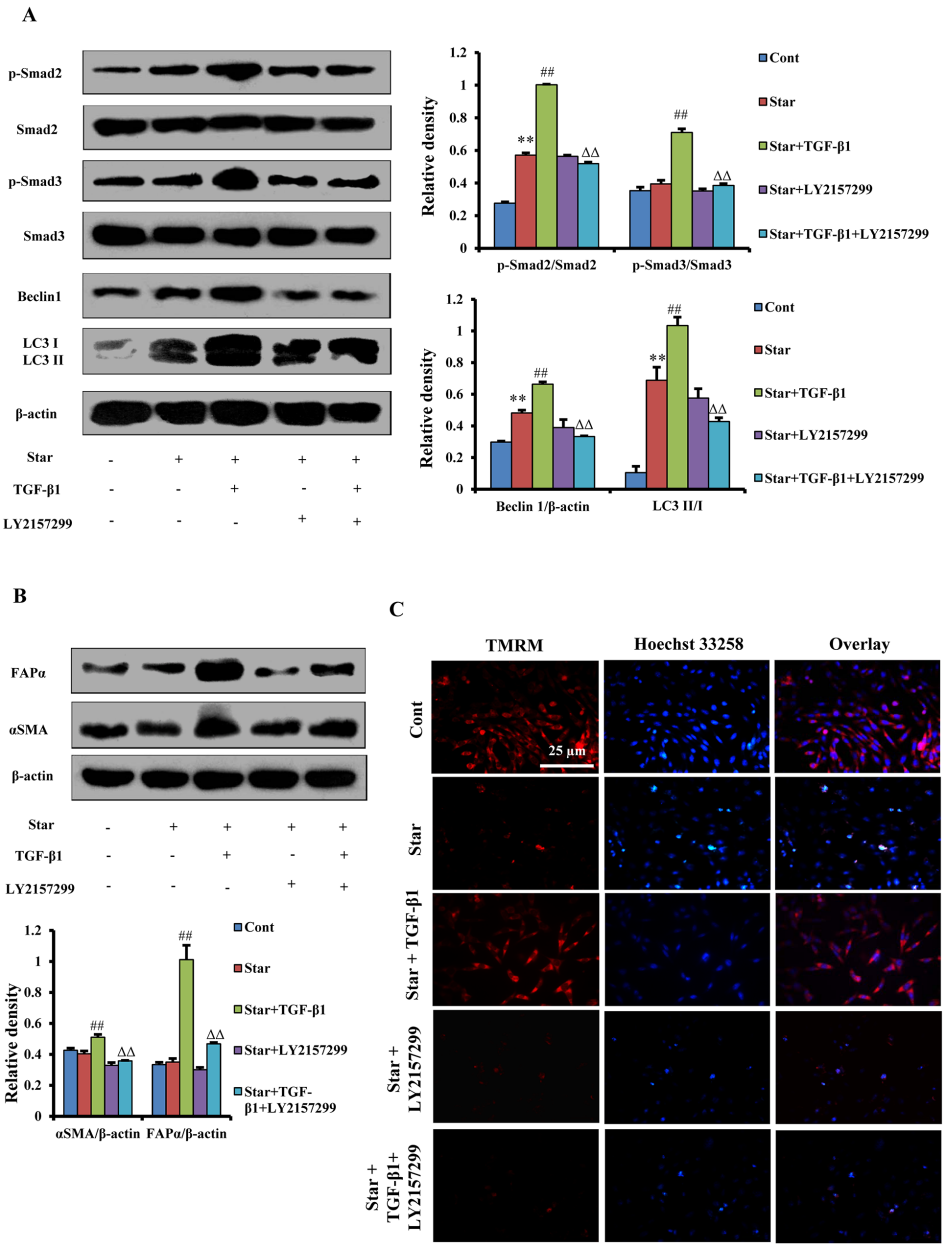
TGF-β/Smad autophagy signaling pathway was involved in TGF-β1-induced protection and formation of CAFs phenotype in Star-treated NIH3T3 fibroblasts Cells were first incubated under free-serum condition for 24 h, followed by treatment in 10% fetal bovine serum with TGF-β1 (2.5ng/ml) in the presence or absence of LY-2157299 (1 μM) for 24 h. **A.** The expression levels of Smads proteins p-Smad2, Smad2, p-Smad3, Smad3 and autophagy-relative proteins Beclin 1 and LC3β-I/II in NIH3T3 fibroblasts were analyzed by Western blotting. **B.** The expression levels of CAFs phenotype α-SMA and FAP-α were analyzed by western blotting. **C.** Evaluation of MMP with TMRM staining using immunofluorescence analysis. Cells were counter-stained with Hoechest 33258 for DNA. Cont, control; Star: starvation, free-serum for 24 h; TGF-β1, 2.5 ng/ml; Rapa, 500 nM; 3-MA, 2 mM; LY-2157299, 1 μM. Data are expressed as the means ± S.E.M. (n=3). ^**^P < 0.01 *vs.* Cont, ^#^*P* < 0.05 and ^##^*P* < 0.01 *vs.* Star-treated cells, and ^ΔΔ^*P* < 0.01 *vs.* Star/TGF-β1 treated group.

It has been reported that Smads were the canonical effectors of TGF-β signaling, which could control autophagy. However, whether Smads proteins have direct transcriptional effects on autophagy remains less clear [30]. To address the issue, we examined the expression levels of p-Smad2, Smad2, p-Smad3, Smad3 and autophagy-relative proteins by western blotting. Our results showed that TGF-β1 up-regulated p-Smad2, p-Smad3, Beclin 1 and LC3β-II/I conversion in Star-treated NIH3T3 fibroblast, but TGF-βR1/ALK5 inhibitor LY-2157299 suppressed the upregulation effects of TGF-β1 (Figure 6A). LY-2157299 further suppressed TGF-β1-induced up-regulation of α-SMA and FAP-α protein expression in Star-treated NIH3T3 fibroblast (Figure 6B). In addition, we evaluated MMP with TMRM staining. Our results showed that LY-2157299 abolished TGF-β1-induced protective effect on mitochondria in Star-treated NIH3T3 fibroblast (Figure 6C). These data suggested that TGF-β1 induced protection and formation of CAFs phenotype was through TGF-β/Smad autophagy signaling pathway.

### TGF-β1-induced autophagy could also activate the formation of CAFs phenotype in tumor microenvironment of mixed xenograft tumor

**Figure 7:**
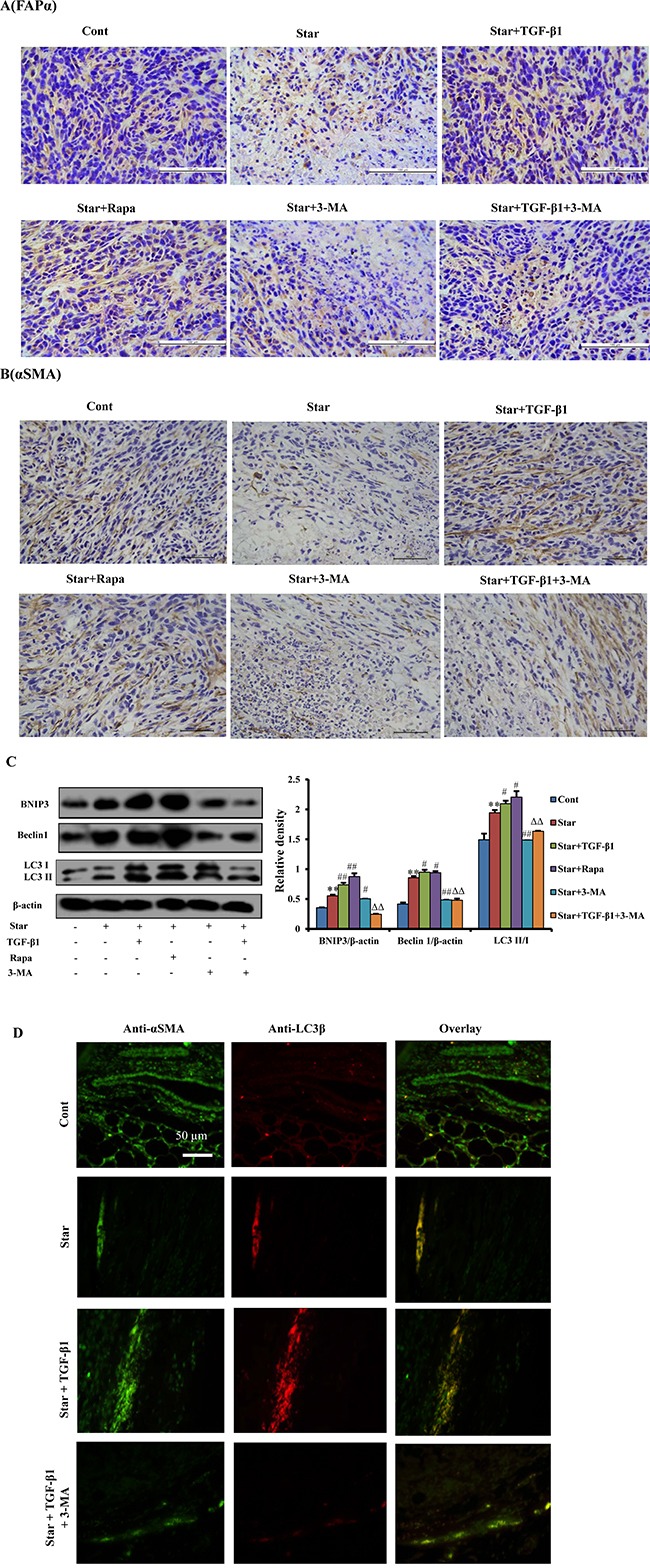
TGF-β1-induced autophagy could also activate the formation of CAFs phenotype in tumor microenvironment of mixed xenograft tumor Cells were first incubated under free-serum condition for 24 h, followed by treatment in 10% fetal bovine serum with TGF-β1 (2.5ng/ml). The treated NIH3T3 fibroblasts were mixed with 4T1 breast cancer cells, and then implanted subcutaneously (s.c.) into the right flank of female Balb/c mice. **A, B.** To evaluate if TGF-β1 induced CAFs features in tumor microenvironment, tumor tissues were analyzed by immunohistochemical staining with antibodies directed against α-SMA and FAP-α. **C.** The protein expression levels of the mitophagy (BNIP3) and autophagy markers [Beclin-1 and LC3II (lower band)] were evaluated by western blotting analysis in tumor tissues. β-actin was used as an equal loading control. **D.** The co-localization of α-SMA and LC3β-II in tumor tissues dually stained with α-SMA (probed with primary anti-α-SMA antibody, and a secondary antibody using FITC 488, Santa Cruz, USA) and LC3β (probed with primary anti-LC3β antibody and a secondary antibody using DyLight 649, Abbkine, USA). Cont, control; Star: starvation, free-serum for 24 h; TGF-β1, 2.5 ng/ml; Rapa, 1 mg/kg; 3-MA, 15 mg/kg. Results presented are the means ± S.E.M. (n=7). ^**^P < 0.01 *vs.* Cont, ^#^*P* < 0.05 and ^##^*P* < 0.01 *vs.* Star-treated cells, and ^ΔΔ^*P* < 0.01 *vs.* Star/TGF-β1 treated group.

It is well known that myofibroblasts or CAFs is essential components in the tumor microenvironment. Our previous research has demonstrated that mixed xenograft tumor model with the ratio of 1:2 (4T1 breast cancer cells: NIH3T3 fibroblast cells) is beneficial to tumor growth (Supplement 1). In order to investigate whether TGF-β1 could induce CAFs features in tumor microenvironment, we used the mixed xenografted Balb/c mice as an *in vivo* model. Our results showed that treatment of TGF-β1 to starved NIH3T3 cells in the mixed xenograft tumor could increase the expression of both CAFs markers, while 3-MA treatment reduced the effects of TGF-β1 (Figure 7A and 7B). These results indicated that TGF-β1 promoted *in vivo* CAFs transformation.

In order to confirm that autophagy was involved in TGF-β1 induced CAFs transformation in tumor microenvironment, we further evaluated the expression of autophagy related protein by western blotting. It was found that the occurrence of autophagy was significantly higher in NIH3T3 fibroblasts with Star treatment in the mixed xenograft tumor. TGF-β1 pro-treatment in Star-treated NIH3T3 fibroblasts significantly enhanced the expression of BNIP3, Beclin 1 and LC3β-II/I conversion in the mixed xenograft tumor, while 3-MA suppressed the effects of TGF-β1 (Figure 7C). Moreover, the co-localization of α-SMA and LC3β-II puncta was promoted by TGF-β1 treatment in the mixed xenograft tumor. In contrast, autophagy inhibitor 3-MA resulted in decreased formation of α-SMA and LC3β-II foci induced by TGF-β1 treatment (Figure 7D).

### TGF-β1-induced autophagy promoted tumor growth of mixed xenograft tumor in Balb/c mice

**Figure 8:**
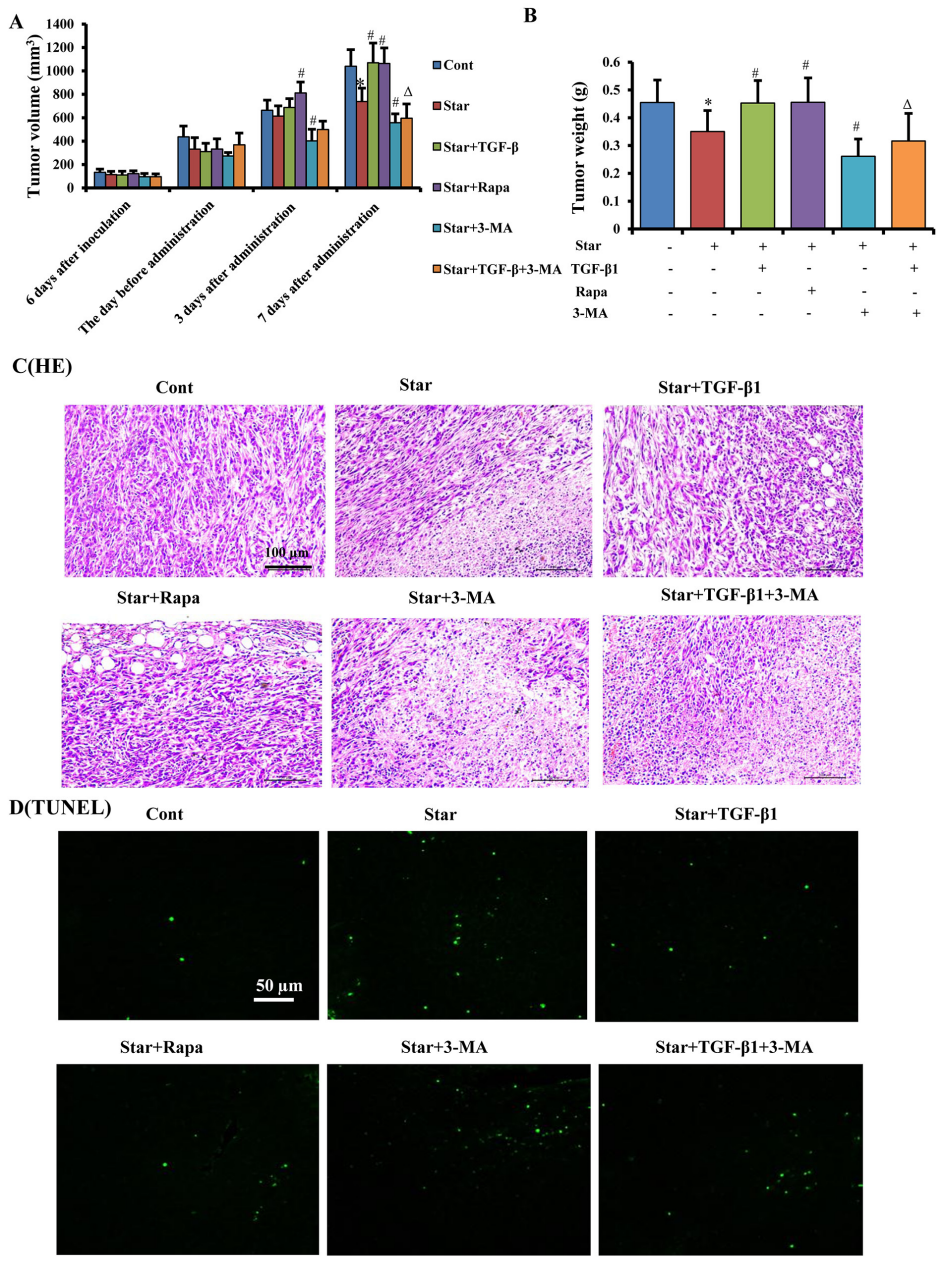
TGF-β1-induced autophagy promoted tumor growth of mixed xenograft tumor in Balb/c mice Cells were first incubated under free-serum condition for 24 h, followed by treatment in 10% fetal bovine serum with TGF-β1 (2.5ng/ml). The treated NIH3T3 fibroblasts were mixed with 4T1 breast cancer cells, and then implanted subcutaneously (s.c.) into the right flank of female Balb/c mice. **A.** Tumor volume was monitored every day by two-dimensional measurements of individual tumors for each mouse. Tumor volume (cm^3^) was calculated according to the formula: (π/6) × tumor length × tumor width^2^. **B.** The tumors were taken 22 days after implantation. The tumor weights were recorded. **C.** Tumor sections were stained with H&E for histological examination to determine tumorous morphology and architectural changes. **D.** Apoptosis of tumor tissues were measured by TUNEL staining. Cont, control; Star: starvation, free-serum for 24 h; TGF-β1, 2.5 ng/ml; Rapa, 1 mg/kg; 3-MA, 15 mg/kg. Results presented are the means ± S.E.M. (n=7). Results presented are the means ± S.E.M. (n=7).^**^P < 0.01 *vs.* Cont, ^#^*P* < 0.05 and ^##^*P* < 0.01 *vs.* Star-treated cells, and ^ΔΔ^*P* < 0.01 *vs.* Star/TGF-β1 treated group.

Our findings have indicated that TGF-β1-induced autophagy triggered formation of CAFs phenotype in tumor microenvironment. To evaluate these effects of TGF-β1 on tumor development *in vivo*, we detected tumor volume and weight in Balb/c mice challenged with mixed xenograft tumor. Our results showed that treatment of NIH3T3 cells with Star suppressed tumor growth in mice, while TGF-β1 pro-treatment in Star-treated NIH3T3 fibroblasts could promote tumor growth. However, the beneficial effects of TGF-β1 were antagonized by 3 days or 7 days of 3-MA (15 mg/kg) administration (Figure 8A and 8B). Furthermore, significant necrosis was found in Star group, while TGF-β1 and Rapa (1 mg/kg) obviously restored the Star-related necrosis, the effect of TGF-β1 was suppressed by 3-MA (Figure 8C). Moreover, TUNEL results showed that apoptotic ratio was significantly higher in Star group. TGF-β1 and Rapa treatment reduced Star-induced apoptosis, while 3-MA could abolish the effect of TGF-β1 treatment (Figure 8D).

## DISCUSSION

In tumor microenvironment, CAFs usually mediate tumor initiation, progression, and metastasis. It can arise from tumor stroma where TGF-β is thought to promote the differentiation of fibroblasts to CAFs [32]. In turn, CAFs secrete large amounts of TGF-β, which induces an autocrine signaling loop and maintains the differentiation of fibroblasts into CAFs [33]. In our study, our results revealed a positive association between TGF-β expression and CAFs phenotype in tumor microenvironment of breast cancer patients. To further investigate the relationship between TGF-β expression and formation of CAFs in tumor microenvironment, we employed starved NIH3T3 fibroblasts *in vitro* and 4T1 cells mixed with NIH3T3 fibroblasts xenograft tumor model *in vivo* to simulate nutritional deprivation of tumor microenvironment.

It has been reported that nutrient deprivation or energy restriction decrease tumor growth by reducing ageing-associated inflammation, elevating glucocorticoid hormone, and decreasing angiogenesis [34]. Moreover, Sanchez *et al.* reported that stromal cells in the nutrient-deprived core utilized autophagy to support the surrounding cells [35]. In our study, we had confirmed that Star led to proliferation inhibition, loss of MMP and cell apoptosis, which were attenuated by TGF-β1 treatment. It is worth noting that TGF-β1 also induced an upregulation of CAFs markers, α-SMA and FAP-α, in Star-treated NIH3T3 fibroblasts. These data suggested that TGF-β1 could protect NIH3T3 cells from nutrient deprivation and induce the formation of CAFs, which were consistent with a previous research on TGF-β1-induced transformation of NIH3T3 fibroblasts to myofibroblasts [5].

Recently, a growing amount of evidence argues for the function of autophagy in tumor microenvironment [36–38]. The activation of autophagy may be a viable therapeutic option for cancer cells located in the core of solid tumors with a nutrient-deficient microenvironment [39]. Moreover, cells survivals depend on autophagy during nutrient deprivation [40]. It has reported that TGF-β1 can induce autophagy to support cell survival in mouse mesangial cells [29] and reduce apoptosis in hepatic stellate cells [41]. In agreement with the previous findings, MDC results showed that Star-induced autophagy was enhanced by TGF-β1 in NIH3T3 fibroblasts, which was found to be prevented by autophagy inhibitors such as wortmannin or 3-MA [42]. Meanwhile, the expression of autophagy related genes and proteins BNIP3, Beclin 1 and conversion of LC3 I to II, indicated that TGF-β1 enhanced Star-induced autophagy. Our TEM and confocal fluorescence imaging data also clearly indicated that TGF-β1 promoted the mitophagy. All of the above confirmed that Star-induced autophagy might be a stress-evoked compensatory mechanism, which was insufficient to fight against Star-induced cellular damages during nutrient deprivation. However, TGF-β1 enhanced Star-induced autophagy and caused protective effects and formation of CAFs in Star-treated NIH3T3 fibroblasts. Autophagy inhibitor 3-MA, *ATG5* siRNA or LY-2157299 could all block this enhancement of autophagy and the protection and CAFs phenotypes formation. These evidences suggested that TGF-β/Smad autophagy was involved in the protective mechanisms and formation of CAFs phenotype by TGF-β1 in Star-treated NIH3T3 fibroblasts. Meanwhile, our results *in vivo* also confirmed that TGF-β1 could induce the formation of CAFs phenotype formation by autophagy in tumor microenvironment of mixed xenograft tumor.

It has been reported that autophagy limits tumor formation in the early stage, while favors tumor cell survival and invasion as soon as cancer is formed [43]. Meanwhile, high stromal autophagy can promote cancer progression [44]. Therefore, TGF-β1-induced autophagy in breast cancer might be implicated in tumor promotion [45]. Our results demonstrated that TGF-β1 treatment effectively prevented Star-induced suppression on tumor growth, while 3-MA prevented the beneficial effects of TGF-β1. Furthermore, TGF-β1 could restore Star-induced necrosis and apoptosis in tumor microenvironment. Taken together, these data demonstrated that TGF-β1-induced autophagy contributed to the formation of CAFs phenotype, and then promoted tumor growth.

In conclusion, we found that TGF-β1 promoted the survival of starved NIH3T3 fibroblasts, and activated the formation of CAFs phenotype in the tumor microenvironment, which could promote tumor growth. It is noteworthy that autophagy inhibitor 3-MA, *Atg5* siRNA or LY-2157299 blocked TGF-β1-induced protective effects and the formation of CAFs phenotypes, suggesting that TGF-β1-induced these effects through TGF-β/Smad autophagy signaling pathway. Moreover, it has been reported that CAFs create a nutrient-rich microenvironment, to metabolically support tumor growth, via the local stromal generation of mitochondrial fuels (lactate, ketone bodies, fatty acids, glutamine, and other amino acids) [46] (Schema 1). The autophagy mechanisms of TGF-β1, thus, make it a unique, potential and more useful to induce formation of CAFs phenotype to promote tumor growth. Therefore, TGF-β1-induced autophagy is a significant determinant in tumor growth and progression for breast cancer therapies.

## MATERIALS AND METHODS

### Reagents

Recombinant human TGF-β1 protein was bought from Peprotech (Rocky Hill, NJ, USA). MitoTracker^®^ Red CMXRos (Mito Red) was bought from Invitrogen (Carlsbad, CA, USA). 4′,6-diamidino-2-phenylindole (DAPI), Hoechst 33258, 3-Methyladenine (3-MA), monodansylcadaverine (MDC), 3-(4,5-dimetrylthiazol-2-yl)-2,5- diphenyltetrazolium bromide (MTT), rapamycin (Rapa) and tetramethylrhodamine methyl ester (TMRM) were purchased from Sigma Chemical (St. Louis, MO, USA). Transforming growth factor-β type I receptor kinase (ALK5) inhibitor (TGF-βR1/ALK5) was purchased from Selleck Chemicals (Houston, Texas, USA). FITC goat anti-mouse IgG was purchased from Santa Cruz Biotechnology (Santa Cruz, CA, USA). Dylight488 goat anti-rabbit IgG and Dylight649 goat anti-rabbit IgG were purchased from Abbkine (Redlands, CA, USA). Polyclonal antibodies against FAP-α, α-SMA, Beclin 1, LC3β, BNIP3, ATG5, p-Smad2, Smad2, p-Smad3, Smad3, caspase-3, caspase-9, Bax, Bcl-2, β-actin and horseradish peroxidase-conjugated secondary antibodies were purchased from Cell Signaling Technology (Beverly, MA, USA). PrimeScript^®^ RT reagent Kit and SYBR^®^ Premix Ex Taq™ were products of TaKaRa (Liaoning, China). Terminal deoxynucleotidyl transferase-mediated dUTP-biotin nick end labeling (TUNEL) Detection Kit was purchased from Beyotime Institute of Biotechnology (Jiangsu, China). AnnexinV–FITC and propidium iodide (Annexin V/PI) apoptosis detection kit was bought from Beyotime Institute of Biotechnology (Jiangsu, China). Small interfering siRNA targeting *ATG5* or non-targeting negative control were purchased from Ribobio (Guangzhou, China).

### Animals

Female Balb/c mice (15-18 g) were purchased from Laboratory Animal Center of Sun Yat-sen University (Guangzhou, China), with Permission No. SYXK 2012-0117. All mice were housed in a room at a mean constant temperature (23 ± 2°C) with a 12-h light-dark cycle, 50-60% relative humidity and free access to standard pellet chow and water. Mice were maintained in these facilities for at least 1 week before experiment. All animal care and experimental procedures were approved by the Animal Care and Use Committee of Laboratory Animal Center of Jinan University (Approval ID: SCXK 2011-0029), and were in accordance with the National Institute of Health's Guide for the Care and Use of Laboratory Animals.

### Cell culture

The NIH3T3 mouse embryonic fibroblasts were obtained from American Type Culture Collection (ATCC, Manassas, VA, USA). Cells were cultured in DMEM medium (Gibco, Grand Island, NY) supplemented with 10% heat inactivated fetal bovine serum (Gibco), 2 mM _L_-glutamine (Gibco), 100 U/mL penicillin and 100 μg/mL streptomycin (Gibco) at 37°C with 5% CO_2_. The cultures in exponential phase were used in the experiments.

### Experimental procedures

Primary tumors were induced by subcutaneous injection of 4T1 breast cancer cells mixed with NIH3T3 fibroblasts in ratio of 1:2 into the right flank of female Balb/c mice. All animals were randomly divided into six groups (n=7): 1. Cont (normal NIH3T3 cells were incubated with 10% serum for 24 h and mice received daily i.p. normal saline), 2. Star (NIH3T3 fibroblasts were incubated with free serum for 24 h and mice received daily i.p. normal saline), 3. Star + TGF-β1 (NIH3T3 fibroblasts were incubated with free serum for 24 h, then treated with 2.5 ng/ml TGF-β1 in 10% serum for an extra 24 h and mice received daily i.p. normal saline), 4. Star + Rapa (NIH3T3 fibroblasts were incubated with free serum for 24 h and mice receive daily i.p.1 mg/kg of Rapa), 5. Star + 3-MA (NIH3T3 fibroblasts were incubated with free serum for 24 h and mice received daily i.p. 15 mg/kg of 3-MA), 6. Star + TGF-β1 + 3-MA (NIH3T3 fibroblasts were incubated with free serum for 24 h, then treated with 2.5 ng/ml TGF-β1 in 10% serum for an extra 24 h and mice received daily i.p. 15 mg/kg of 3-MA). Seven days after tumor growth was monitored every day by two-dimensional measurements of individual tumors for each mouse. Two weeks after reagent was administrated each day for seven days. Tumor volume (cm^3^) was calculated according to the formula: (π/6) × tumor length × tumor width^2^. At the end of the experiment (day 22 after tumor implantation), the animals were sacrificed. Tumors were excised before determinations of biochemical parameters.

### MTT assay

The growth inhibitory effect of reagents on cells was measured by MTT assay. Cells were dispensed in 96-well plate at a density of 1 × 10^5^ cells per well. After 24h incubation, cells were treated with the tested agents for the indicated periods of time. A 20 μl aliquot of 0.5% MTT solution was added to each well followed by 4h incubation. Optical density was measured using an ELISA reader (Thermo Fisher Scientific, Franklin, MA, USA). Statistical significance of the experimental data from three independent experiments was determined by the Student's t-test, and *P* values < 0.05 were considered significant.

### Histological Examination

For histological evaluation, all tumor samples were fixed in formalin, embedded in paraffin, and 4 *μ*m thick sections were prepared and stained with hematoxylin and eosin (H&E). These specimens were observed under a light microscope.

### Immunohistochemical Staining

Tumor sections (4 *μ*m) were dewaxed and rehydrated regularly and then treated with 3% H_2_O_2_. The sections were then pretreated with a microwave antigen retrieval technique. Then the non-specific sites were blocked with 10% goat serum for 30min at room temperature. The specimens were then incubated overnight at 4°C with the following antibodies: anti-α-SMA (1: 500), anti-FAP-α (1:500) and anti-TGF-β (1:500). On the second day, after incubation with secondary antibody, an antibody binding analysis was performed using a DAB kit. Finally, the slides were counterstained with hematoxylin and observed under a light microscope.

### TUNEL assay

TUNEL assay for fluorescence microscopy was conducted following the instruction of the detection kit. Tumor samples acquired were fixed in 4% paraformaldehyde overnight and embedded with paraffin. Paraffin blocks of tumor were cut into 4 *μ*m slices and then processed using standard deparaffinization and rehydration techniques. Slices were incubated in PBS containing 0.1% Triton X-100 for 2 min. Afterwards, slices were washed twice with PBS and stained with TUNEL detection fluid for 1 h, and then washed three times with PBS. FITC-labeled TUNEL-positive cells were imaged by Leica-CTR MIC fluorescent microscope (Leica Camera AG, Solms, Germany) under 488-nm excitation and 530-nm emission. Microscopic photographs were merged by Adobe Photoshop CS3 Extended software (version: 10.0.1; Adobe, San Jose’, CA, USA).

### Q-PCR (Quantitative Real-Time PCR) assay

Total mRNA of the cells was extracted after treatment of the indicated time. First strand cDNA synthesis was generated from 500 ng of total RNA. Quantification of target and reference (β-actin) genes was performed in triplicate on LightCycler^®^ 480 II (Roche, Applied Science). The primers used in each reaction were as follows: BECN1 forward 5′ TTACCACAGCCCAGGCGAAA 3′ and reverse 5′ TCCCCGATCAGAGTGAAGCTATTAG 3′; LC3β forward 5′ TGTAGGATATAGCTCTAAGCCGGGT 3′ and reverse 5′ TCAGCAGAAGGGCGTATGGTAAC 3′; β-actin forward 5′ TGAGAGGGAAATCGTGCGTGAC 3′ and reverse 5′ GCTCGTTGCCAATAGTGATGACC 3′. After normalized to β-actin gene, expression levels for each target gene were calculated using the comparative threshold cycle (CT) method. The Δct values were calculated according to the formula Δct = ct (gene of interest)-ct (β-actin) in correlation analysis, and the 2-ΔΔct was calculated according to the formula ΔΔct = Δct (control group) -Δct (experimental group) for determination of relative. Data are presented as the mean standard deviation (SD) from three independent experiments.

### Western blotting analysis

Following various treatments, cells were resuspended in lysis buffer (Beyotime Institute of Biotechnology) on ice for 5 min, and the supernatants were collected after centrifugation at 13,000 × *g* for 15 min. Protein lysates (30 μg) were separated in 10% or 15% SDS-PAGE and blotted onto nitrocellulose membrane (Amersham Biosciences, Piscataway, NJ, USA). Proteins expression were detected using polyclonal antibody and visualized using anti-rabbit and anti-mouse IgG conjugated with horseradish peroxidase (HRP) and Pierce^®^ ECL Western Blotting Substrate (Thermo Fisher Scientific) as the substrate of HRP.

### TMRM assay for mitochondrial membrane potential (MMP) using flow cytometer

MMP (Δψ_m_) was measured with flow cytometry using TMRM staining. TMRM accumulates in normal mitochondria due to its positive charge whereby the reduction of MMP leads to the release of TMRM. Following various treatments, cells were incubated with 100 nM TMRM at 37°C for 30 min. Harvested cells were immediately analyzed for potential using Beckman Coulter Epics XL flow cytometer (650 nm long pass filter) equipped with Expo32 ADC.

### Assessment of MMP and apoptosis using fluorescent microscopy

Following various treatments, cells were stained with 20 μM Hoechst 33258 and 100 nM TMRM at 37°C for 30 min. The images were recorded on a Leica CTR MIC fluorescent microscope (Leica Camera AG, Solms, Germany).

### MDC assay for autophagic vacuole

To confirm the occurrence of autophagy, MDC was used as it can selectively incorporate into autophagosomes and autolysosomes. Following various treatments, cells were incubated with 10 μM MDC at 37°C for 1 h, intracellular MDC fluorescence (530/30 nm bandpass filter) was determined using Leica CTR MIC fluorescent microscope (Leica Camera AG, Solms, Germany) within 30 min after incubation.

### Assessment of autophagy

Following various treatments, NIH3T3 cells were stained with MitoRed at 37°C for 30 min, and then fixed in 4% paraformaldehyde. Samples were labeled with anti-LC3β at 4°C overnight and incubated with secondary antibody-Alexa flour 488^®^ at 37°C for 4 h. Samples were counterstained with DAPI before being imaged using a Leica CTR MIC fluorescence microscope or Zeiss LSM510 Meta DuoScan laser scanning confocal microscope (Carl Zeiss AG, Oberkochen, Germany) as indicated.

### Assessment of co-localization of α-SMA and LC3β

Following various treatments, NIH3T3 cells were fixed in 4% paraformaldehyde. Samples were labeled with anti-α-SMA and anti-LC3β at 4°C overnight and incubated with secondary antibody- FITC 488 and DyLight 649 at 37°C for 2 h. Samples were counterstained with DAPI before being imaged using a Leica CTR MIC fluorescence microscope or Zeiss LSM510 Meta DuoScan laser scanning confocal microscope (Carl Zeiss AG, Oberkochen, Germany) as indicated.

### Transmission electron microscopy (TEM) for observation of autophagic ultrastructure

Following various treatments, NIH3T3 cells were fixed with 2 % glutaraldehyde in 0.1 M PBS (pH 7.3) for 2 h at 4°C and washed extensively with 0.1 M cacodylate buffer including 0.1 % CaCl_2_. The samples were fixed in 0.1 M cacodylate buffer including 0.1 % CaCl_2_ for at least 30 min and then dehydrated with ethanol series and polymerized at 60°C for 2 days. After being cut by ultracut microtome, the sections were stained with uranyl acetate and lead citrate and analyzed with a Philips Tecnai 10 transmission electron microscope (FEI, Hillsboro, OR, USA).

### *ATG5* Knockdown using siRNA procedure

Small interfering siRNA targeting *ATG5* or non-targeting negative control were purchased from Ribobio (Guangzhou, China). NIH3T3 cells at 50% confluency were transfected for 48 h with siRNA of *ATG5* or control siRNA using Lipofectamine 2000 (Invitrogen), according to the manufacturer's instructions. Transfection efficiency was optimized by trying a range of siRNA and Lipofectamine 2000 concentrations.

### Annexin V/PI Staining analysis

Following various treatments, cells were washed with PBS, stained for AnnexinV–FITC and propidium iodide according to the manufacturer's protocol, and analyzed using Beckman Coulter Epics XL flow cytometer (650 nm long pass filter) equipped with Expo32 ADC.

### Patients and specimen selection

The paraffin-embedded postoperative tissue specimens were obtained from the archives of the Department of Pathology, the Third People's Hospital of Huizhou, between January of 2006 and June of 2012. We retrospectively retrieved 121 tumor specimens of patients with clinical stage I-IV breast cancer. In addition, the paraffin-embedded normal breast tissue specimens (n=10) were obtained from the archives of the Sichuan Provincial Cancer Hospital. The study protocol was approved by the Institutional Ethics Committee of the Third People's Hospital of Huizhou, Sichuan Provincial Cancer Hospital and Jinan University. All participants were acknowledged by providing their written informed consent to participate in this study. Our ethics committees approved this consent procedure.

### Statistics

All data were expressed as means ± S.E.M. of at least three independent experiments. The data were analyzed by ANOVA using Statistics Package for Social Science (SPSS) software (version 19.0; SPSS, Chicago, IL, USA) and LSD-post-hoc test was employed to assess the statistical significance of difference between control and treated groups. In case *P* < 0.05 was considered statistically significant.

## SUPPLEMENTARY FIGURES



## Abbreviations

TGF-β1: transforming growth factor-β1
CAFs: cancer-associated fibroblasts
Star: serum starvation
MMP: mitochondrial membrane potential
Rapa: rapamycin
3-MA: 3-methyladenine
α-SMA: alpha-smooth muscle actin
FAP-α: fibroblast activation protein-alpha
Smad2: mothers against decapentaplegic homolog 2
Smad3: mothers against decapentaplegic homolog 3
DCIS: ductal carcinoma *in situ*
TGF-βR1/ALK5: transforming growth factor-β type I receptor kinase (ALK5) inhibitor
MitoRed: MitoTracker^®^ Red CMXRos
LC3: microtubule-associated protein 1 light chain
Atg: autophagy related protein.
