# Supplementary material for: Autophagy is involved in TGF-β1-induced protective mechanisms and formation of cancer-associated fibroblasts phenotype in tumor microenvironment
Source: Oncotarget. 2015 Dec 21;7(4):4122–41. doi: 10.18632/oncotarget.6702 (PMC4826194; doi:10.18632/oncotarget.6702)
Supplement: Supplementary file 1 [file oncotarget-07-4122-s001.pdf]

SUPPLEMENTARY FIGURES

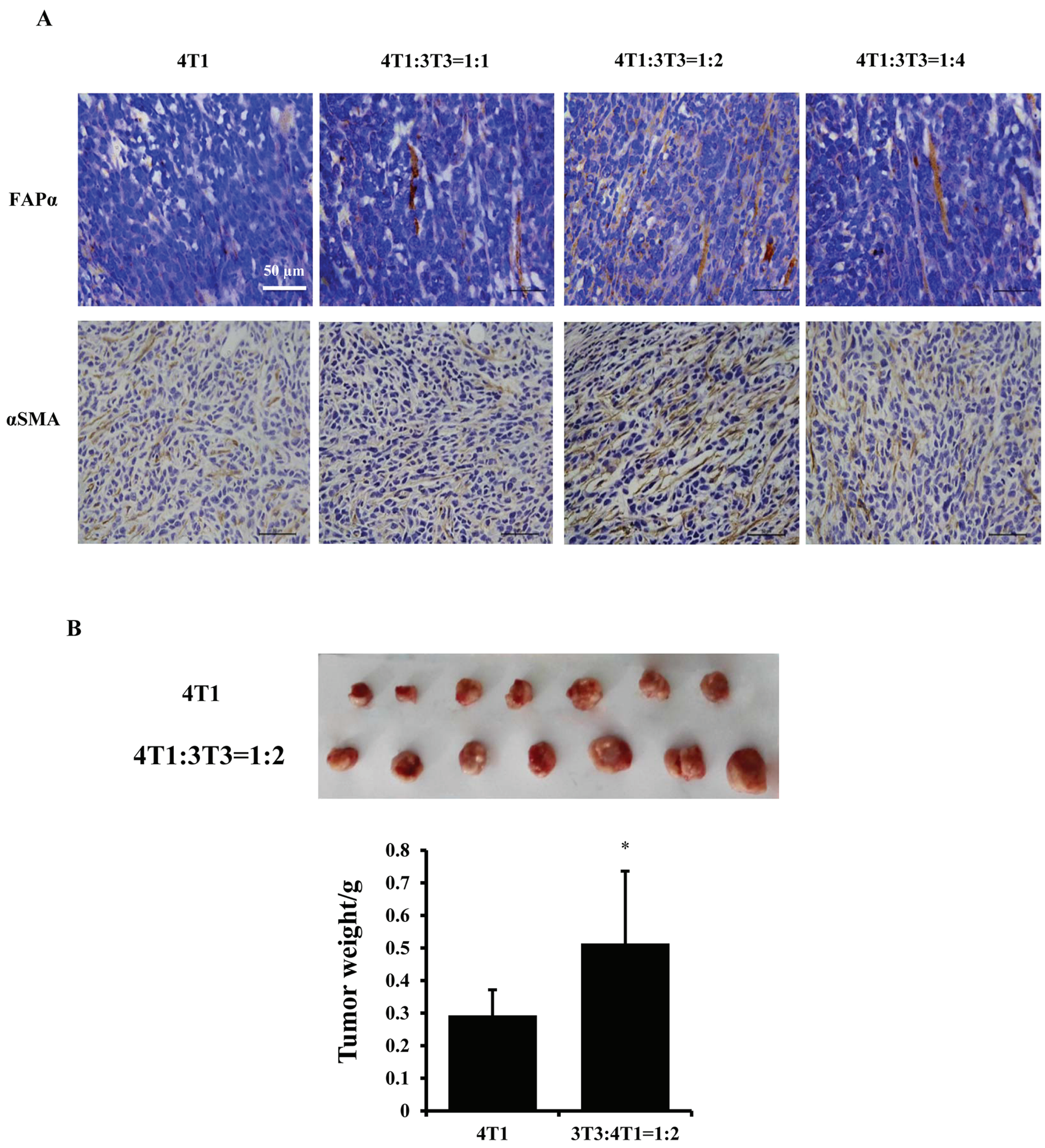

Supplementary Figure S1: The establishment of mixed xenograft model in Balb/c mice. The experiment was carried out by subcutaneously (s.c.) implanting 4T1 breast cancer cells or a mixture of 4T1 cells and serum starved NIH3T3 fibroblasts in Balb/c mice. **A.** Tumor volume was monitored every day by two-dimensional measurements of individual tumors for each mouse. Tumor volume (cm^3) was calculated according to the formula: $(\pi/6) \times$tumor length $\times$ tumor width^2. **B.** The tumors were taken on the 22th day after implantation. Tumor weights were recorded.

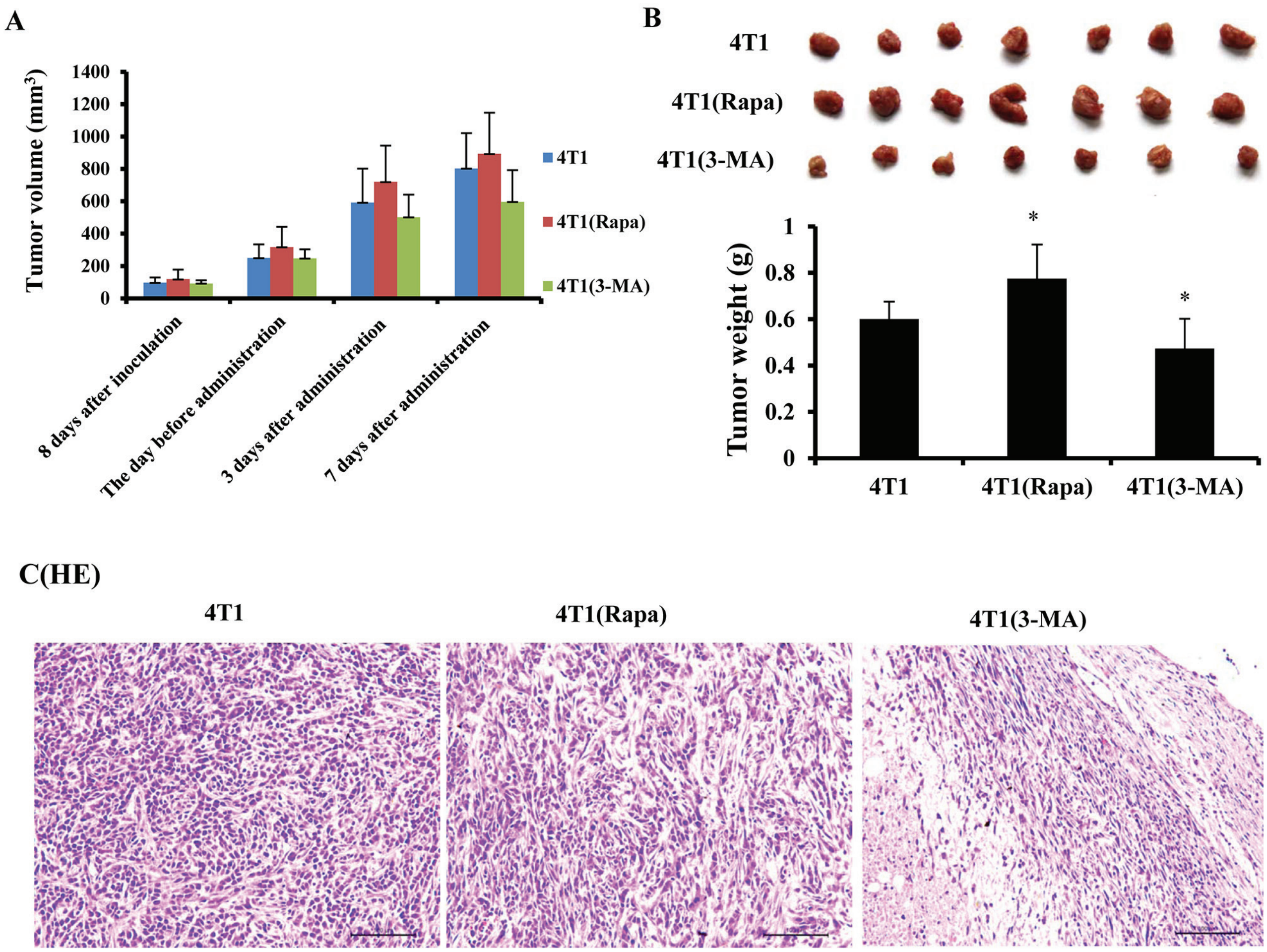

Supplementary Figure S2: Autophagy was involved in TGF-β1 promoted tumor growth in Balb/c mice. 4T1 breast cancer cells were subcutaneously (s.c.) implanted into the right flank of female Balb/c mice. **A.** Tumor volume was monitored every day by two-dimensional measurements of individual tumors each mouse. Tumor volume (cm^3) was calculated according to the formula: (π/6) × tumor length × tumor width^2. **B.** The tumors were taken on the 22th day after implantation. Tumor weights were recorded. **C.** Tumor sections were stained with H&E to determine tumorous morphology and architecture changes. Cont, control; Rapa, 1 mg/kg; 3-MA, 15 mg/kg. Results presented are the means ± S.E.M. (n =7). Results presented are the means ± S.E.M. (n=7). $^*P < 0.01$ *vs*. Cont.

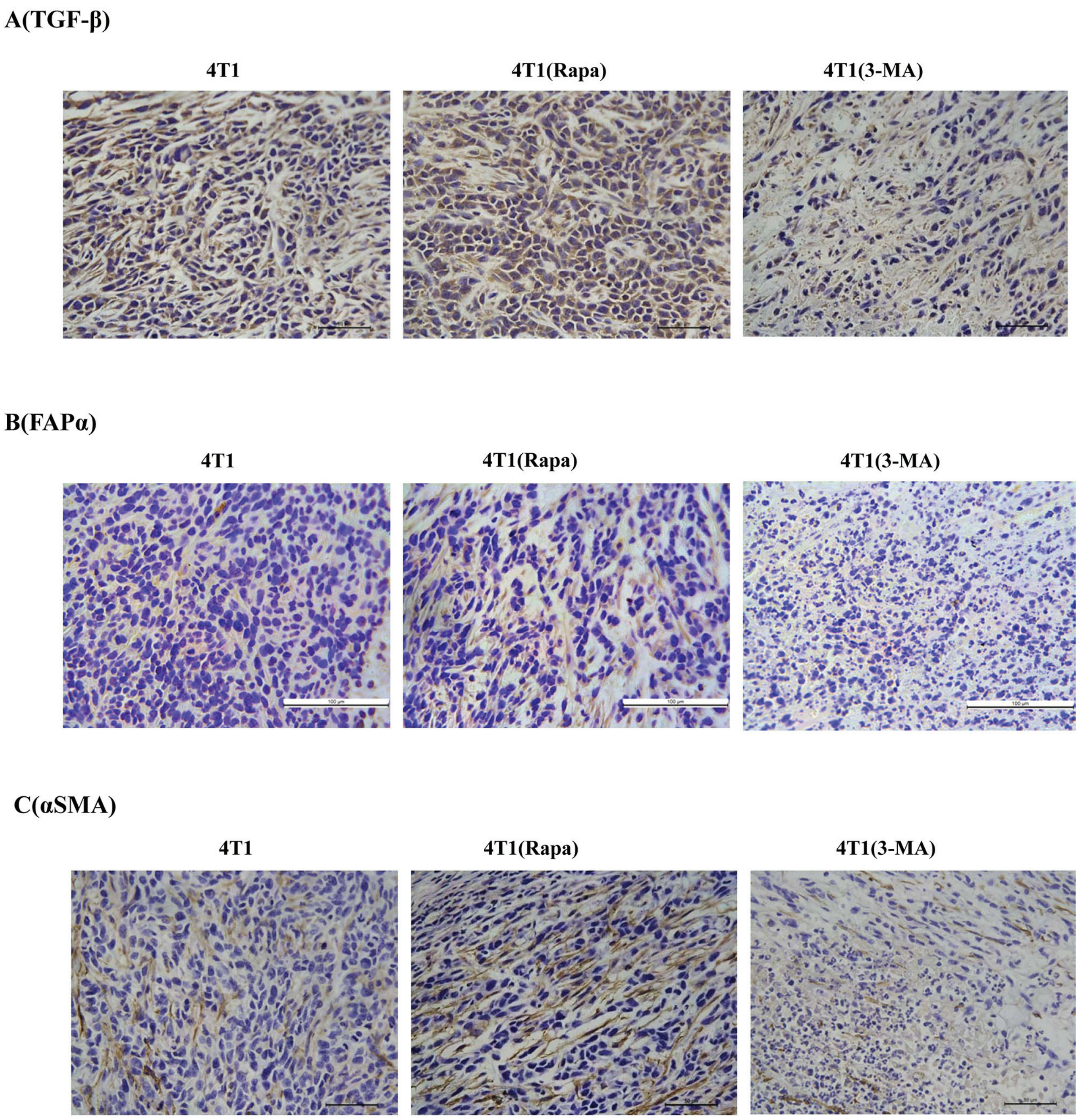

Supplementary Figure S3: Autophagy induced formation of CAFs possibly through TGF-β expression in Balb/c mice. 4T1 breast cancer cells were implanted subcutaneously (s.c.) into the right flank of female Balb/c mice. Tumor tissues were analyzed by immunohistochemical staining with antibodies directed against TGF-β, α-SMA and FAP-Aα.

Note: Our previous study found that autophagy agonist rapamycin promoted breast cancer tumor growth, which was suppressed by 3-MA in 4T1 xenografted BALB/c mice (Supplement 2), suggesting autophagy could promote breast cancer tumor growth. Moreover, rapamycin upregulated the expression levels of TGF-β, α-SMA and FAP-Aα in 4T1 xenograft (Supplement 3), suggesting autophagy induced formation of CAFs possibly by increasing the expression of TGF-β.

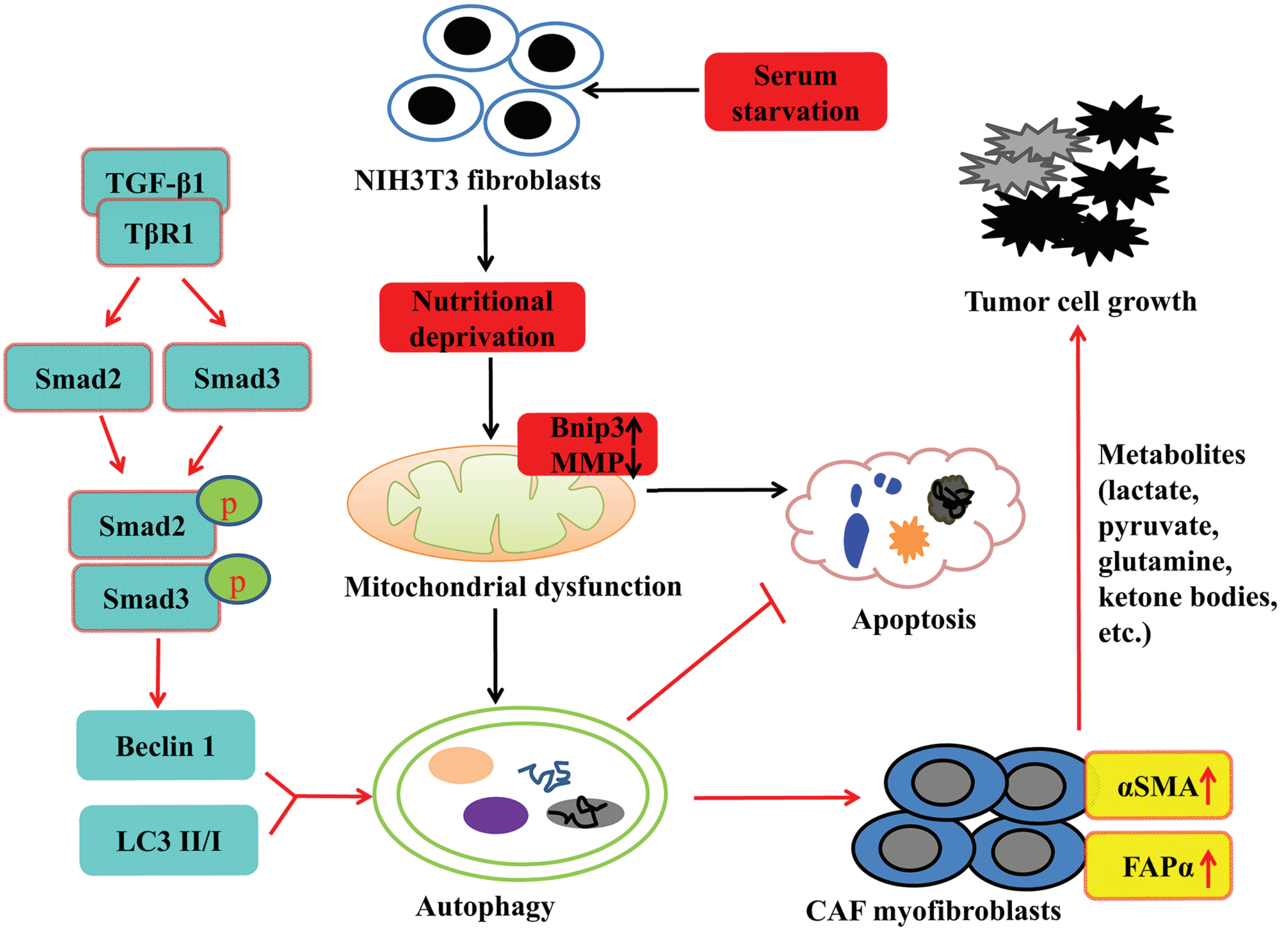

Supplementary Figure S4: Schema 1.
